# 3D Computerised Analysis of Airway Morphology in Subjects From Tamil Nadu: A Pilot Study

**DOI:** 10.7759/cureus.101345

**Published:** 2026-01-12

**Authors:** Aravindh Swamy, Thiruppathy Manigandan, Ramalingam Shakila, K Hariniabilasha

**Affiliations:** 1 Department of Oral Medicine and Radiology, Bharath Institute of Higher Education and Research, Chennai, IND; 2 Department of Oral Medicine and Radiology, Karapaga Vinayaga Institute of Dental Sciences, The Tamil Nadu Dr. M.G.R. Medical University, Padalam, IND; 3 Department of Oral Medicine and Radiology, Sree Balaji Dental College and Hospital, Bharath Institute of Higher Education and Research, Chennai, IND; 4 Department of Orthodontics and Dentofacial Orthopedics, Karapaga Vinayaga Institute of Dental Sciences, The Tamil Nadu Dr. M.G.R. Medical University, Padalam, IND

**Keywords:** airway morphology, cone-beam computed tomography (cbct), obstructive sleep apnea, pharyngeal airway volume, three-dimensional imaging

## Abstract

Introduction: This cross-sectional pilot study aimed to describe and compare age-related variations in airway volume, airway length, smallest airway cross-sectional area, and airway index using a computerised 3D cone-beam computed tomography (CBCT)-based assessment in subjects from the state of Tamil Nadu, India. Age-based comparisons across 11 independent groups were performed to provide descriptive information that may assist in contextualising airway measurements in orthodontic and airway assessment, without implying diagnostic accuracy or causal inference.

Materials and methods: A total of 110 healthy participants aged between six and 60 years were distributed equally across 11 age groups. Cone-beam CT scans were obtained under standard best-practice acquisition protocols. Intergroup comparisons were performed using one-way analysis of variance (ANOVA), and associations between age and airway parameters were evaluated using bivariate regression analysis, based on a statistical significance threshold of p < 0.05. The four dependent variables analysed were airway volume, airway length, smallest airway cross-sectional area, and airway index.

Results: Significant age-group differences were observed for airway volume, smallest airway area, and airway index (p < 0.001). Higher values for these parameters were observed in the 21- to 35-year age group, followed by lower values in older age groups. Airway length demonstrated minimal variation across age groups and did not show statistically significant differences (p = 0.058).

Conclusion: The findings of this cross-sectional pilot study describe age-associated variations in CBCT-derived airway volume, airway length, smallest airway cross-sectional area, and airway index among participants from Tamil Nadu and highlight potential clinical relevance for orthodontic diagnosis and airway assessment, while acknowledging that causal inferences and definitive normative standards cannot be established.

## Introduction

Airway morphology plays an important role in craniofacial structure, respiratory function, and orthodontic treatment planning [1,2]. In conditions such as aberrant facial growth, malocclusion, and sleep-disordered breathing, including obstructive sleep apnea, variations in upper airway morphology have been reported, making airway evaluation a relevant consideration in orthodontics, surgical planning, and clinical assessment [3,4]. Two-dimensional (2D) imaging modalities, including lateral cephalograms, have traditionally been used to assess airway characteristics such as volume, minimal cross-sectional area, length, and airway index; however, these methods are limited by superimposition and lack of 3D accuracy [5,6].

With the increasing use of cone-beam computed tomography (CBCT), 3D assessment of airway morphology has become more feasible in clinical research settings, allowing improved visualization and quantitative description of airway dimensions within defined study samples [7,8]. Despite this advancement, most CBCT-based airway studies have been conducted in Western or East Asian populations [9,10]. Findings from such studies may not be directly applicable to Indian populations due to ethnic, craniofacial, and environmental heterogeneity [11].

True population-based airway studies would require stratified, multistage sampling across multiple geographic regions and inclusion of participants without clinical indications for CBCT imaging. In contrast, most available CBCT investigations, including pilot studies, rely on clinically indicated scans, which may introduce spectrum bias and limit population-level generalizability. Existing CBCT studies from India remain limited in number and are frequently focused on malocclusion-specific or orthodontic patient groups rather than healthy individuals. Additionally, variability in sample characteristics, imaging protocols, and analytical methods limits direct comparison across studies [12]. Consequently, information describing airway morphology across different age groups within regional Indian populations remains sparse.

Rather than establishing population normative standards, cross-sectional pilot investigations are suited to providing descriptive data on age-associated variations and intergroup differences within defined samples [13]. Such data may assist in contextualizing airway measurements in clinical practice and generating hypotheses for future longitudinal research [14,15]. Within this framework, evaluation of CBCT-derived airway measurements across age groups in a regional cohort can offer preliminary insight into age-related patterns without implying population representativeness, causality, or normative reference values.

Accordingly, given the limited descriptive data on CBCT-based airway morphology in subjects from Tamil Nadu, this pilot cross-sectional study was designed to examine age-associated differences in selected airway measurements using computerized 3D CBCT analysis. Each age group consisted of 10 participants, ensuring equal representation across the defined age intervals. The comparisons were intended to explore associations between age and airway morphology within this study sample rather than to generate population norms or developmental benchmarks.

The findings are presented as exploratory reference data that may inform clinical interpretation within similar clinical contexts, guide hypothesis-driven investigations, and support the design of future large-scale, multicentric, population-based, and longitudinal studies employing appropriate sampling frameworks [16-20]. Prior CBCT investigations have demonstrated both the clinical utility of 3D airway assessment and the importance of explicitly reporting measurement reliability when comparing airway volumes across different cohorts, thereby reinforcing the relevance of CBCT-based airway analysis for informed clinical decision-making in orthodontics and airway evaluation [21].

Objectives of the study

The primary objective of this cross-sectional pilot study was to examine the association between age and CBCT-derived airway volume, airway length, smallest airway cross-sectional area, and airway index across eleven independent age groups between six and 60 years. No sex-based comparisons were performed, and the analyses were limited to age-related associations between these four airway dimensions.

## Materials and methods

Study design

The pilot study was a cross-sectional investigation of airway morphology in subjects from Tamil Nadu using computerized 3D analysis. The study was designed to allow comparison across age groups to identify inter-age-group variations in airway structure, an objective appropriate for a cross-sectional design. The sample size was determined pragmatically, consistent with the exploratory nature of a pilot study, to assess feasibility, measurement reliability, and variability of CBCT-derived airway parameters across age groups. Eleven age groups were defined, and approximately 10 participants were included per group to ensure balanced representation and enable preliminary intergroup comparisons, rather than to achieve population-level statistical power.

A total of 110 clinically healthy participants were enrolled, with 10 participants assigned to each of the 11 age groups. Healthy participants were defined as individuals without known craniofacial anomalies, syndromic conditions, chronic respiratory disease, prior orthodontic or orthognathic treatment, or documented airway pathology, and who were undergoing CBCT imaging for clinical indications unrelated to airway disease. Sampling was based on convenience, using clinically indicated CBCT scans available during the study period, rather than random population-based sampling. The CBCT scans provided the imaging data, and volumetric analysis was performed using standardized computer-based assessment methods.

Given the limited sample size within each age group, the precision of the estimated mean airway measurements is limited, and the resulting values should be interpreted as exploratory. The study was not powered to estimate narrow confidence intervals or definitive reference ranges but rather to provide preliminary estimates of variability and trends across age groups to inform future, adequately powered studies. Accordingly, the design enabled structured comparison across age groups to explore age-associated differences in airway dimensions within this study sample, without implying causality or population normative values.

Study population

A total of 110 healthy individuals were recruited and split equally into 11 age brackets, each containing 10 individuals. All CBCT records were retrospectively obtained from the Department of Oral Medicine and Radiology at a university-affiliated dental teaching hospital, where scans were acquired for routine clinical diagnostic purposes. The sample included participants between six and 60 years of age, encompassing childhood, adolescence, and adulthood. The age groups were defined in five-year intervals (six to 10, 11 to 15, 16 to 20, 21 to 25, 26 to 30, 31 to 35, 36 to 40, 41 to 45, 46 to 50, 51 to 55, and 56 to 60 years) to reflect key stages of craniofacial growth, skeletal maturation, and post-maturational stability and decline, as reported in prior airway and craniofacial growth literature. This stratification allowed structured inter-age-group comparisons while maintaining manageable group sizes suitable for a pilot study.

Although both male and female participants were included, sex-specific distribution and comparisons were not analyzed in this pilot study. Similarly, craniofacial skeletal classification and detailed BMI stratification were not recorded or incorporated into the statistical analysis. These factors were therefore not controlled analytically and are acknowledged as important methodological limitations. The sampling strategy was based on convenience sampling of clinically indicated CBCT records from this single academic center rather than population-based random sampling, which may introduce spectrum bias and limit population-level generalizability.

Inclusion criteria

The subjects that participated were medically fit and had no prior history of airway obstruction. Participants had no history of orthodontic or orthognathic treatment. 'Normal nasal breathing' was assessed clinically at the time of CBCT acquisition based on patient history and routine clinical examination, including absence of reported chronic mouth breathing, nasal obstruction, or diagnosed respiratory disorders. No objective functional respiratory tests (such as rhinomanometry or polysomnography) were performed, and nasal breathing was therefore evaluated subjectively, consistent with routine clinical screening in pilot CBCT studies.

The BMI was assessed clinically at the time of patient evaluation using measured height and weight and calculated as weight (kg) divided by height squared (m²). Participants were screened to exclude individuals with clinically apparent underweight or obesity based on age- and sex-appropriate reference ranges; however, BMI values were not recorded as continuous variables nor stratified for statistical analysis. Accordingly, BMI was used only as a screening criterion to minimize extreme weight-related airway variation and was not analyzed as an independent variable. Although individuals with gross craniofacial abnormalities were excluded, detailed craniofacial skeletal classification was not performed. Because BMI and craniofacial pattern were not incorporated into the analytical models, their potential influence on airway dimensions could not be evaluated and is acknowledged as a significant limitation of this pilot study.

Exclusion criteria

Any subject was excluded when there was a craniofacial abnormality, bony deficiency, or syndromic disorder affecting airway morphology. Cases with cleft lip, cleft palate, chronic respiratory disease, prior airway-related procedures, ongoing orthodontic or orthognathic treatment, or systemic diseases affecting craniofacial development were excluded.

Participants receiving long-term medication known to potentially influence airway dimensions or craniofacial soft tissues were also excluded. These included medications prescribed for chronic respiratory conditions (e.g., long-term bronchodilators or inhaled corticosteroids), systemic corticosteroid therapy, long-term muscle relaxants or sedatives, medications affecting neuromuscular control of the upper airway, and long-term hormonal or metabolic therapies that may alter body composition or soft-tissue distribution. Despite these criteria, the absence of detailed skeletal classification and BMI stratification limits comprehensive adjustment for all known airway-related confounders.

Imaging protocol

The CBCT scans were obtained using an i-CAT Next Generation scanner (Imaging Sciences International, Hatfield, PA, USA). Standardized acquisition parameters were followed, including a 13 × 16 cm field of view, 0.25 mm voxel size, 120 kVp tube voltage, 5 mA tube current, and a scan time of 40 seconds, consistent with established CBCT airway imaging protocols [1-3,7,15]. Subjects were positioned upright with the Frankfurt horizontal plane parallel to the floor, teeth in maximum intercuspation, and tongue relaxed. All scans were acquired by a single trained radiographer to ensure consistency, and the resulting Digital Imaging and Communications in Medicine (DICOM) data were exported for volumetric airway analysis following validated CBCT methodologies [2,6,7]. All CBCT scans were clinically indicated, and no scans were acquired solely for research purposes.

Airway analysis

The Invivo 5 software (Anatomage, Santa Clara, CA, USA) was used to perform airway segmentation and volumetric analysis. This is commercially licensed software accessed under the institution’s subscription. Semi-automatic 3D reconstructions of the upper airway were generated from the posterior nasal spine (PNS) superiorly to the inferior border of the fourth cervical vertebra (C4), following previously validated CBCT airway analysis protocols [6,17]. Four airway-related variables were measured: average smallest airway area (mm²), airway length (mm), airway volume (mm³), and the volume-to-length ratio defined as the airway index, based on established airway measurement guidelines [1,7,15].

For airway segmentation, a standardized gray-scale threshold range recommended by the Invivo 5 software for upper airway analysis was applied uniformly across all CBCT scans. Semi-automatic region-growing segmentation was performed using identical software settings for all cases, followed by slice-by-slice visual inspection and manual refinement where necessary to ensure accurate delineation of airway boundaries and exclusion of adjacent soft tissue structures. All segmentations were carried out using consistent anatomical definitions and segmentation parameters to maintain methodological reproducibility across the sample.

**Figure 1:**
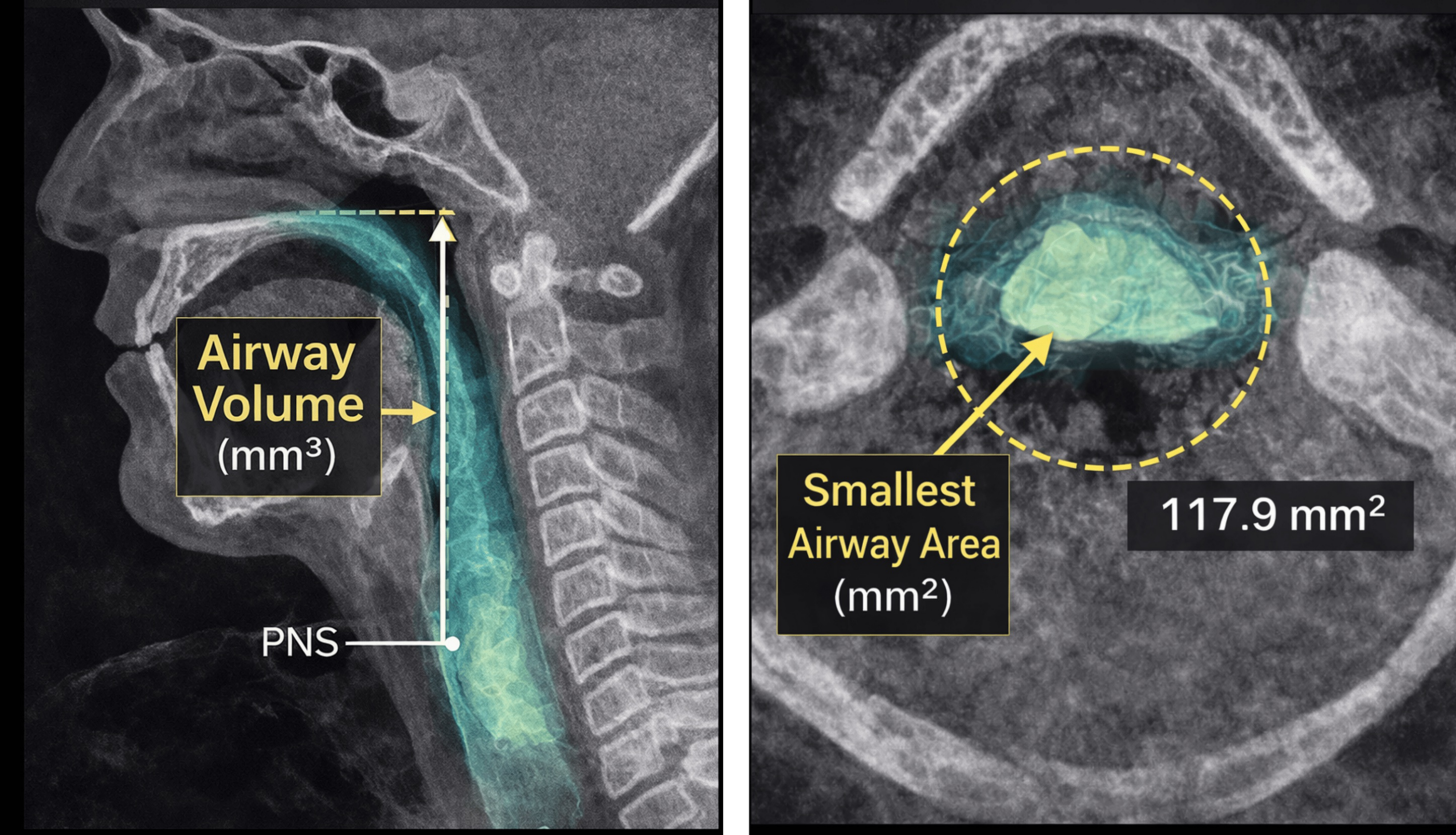
CBCT-based airway measurement illustration CBCT: Cone-beam computed tomography

**Figure 2:**
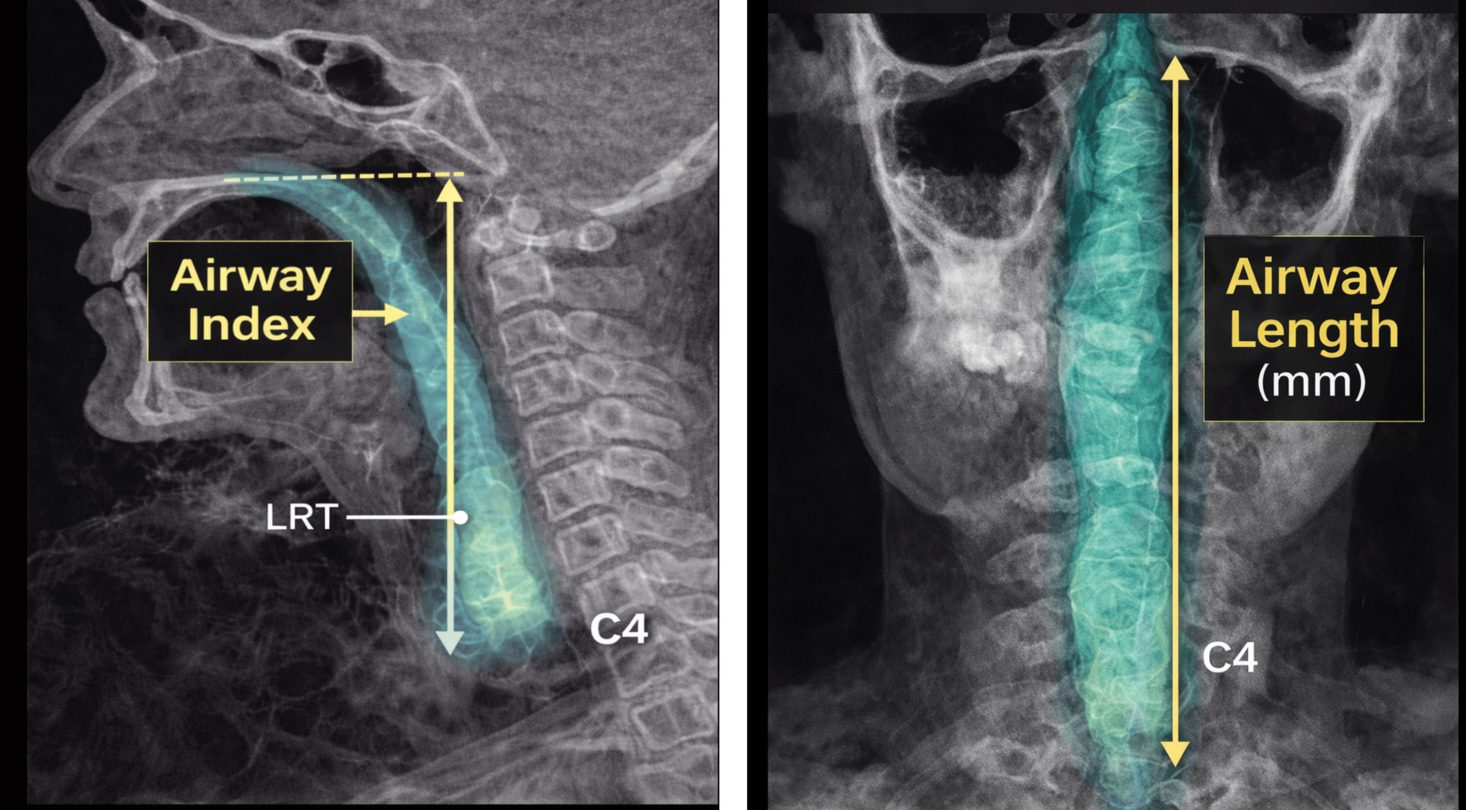
CBCT depiction of airway length and airway index measurement landmarks CBCT: Cone-beam computed tomography

Two measurements were performed by the same calibrated examiner to assess intra-observer reliability, and a subgroup of scans was independently re-measured by a second examiner to evaluate inter-observer reliability using standard reliability assessment methods [6,17]. Examiner calibration was completed prior to data collection through repeated measurements on a subset of scans until acceptable intra-observer agreement was achieved. For reliability assessment, 15 randomly selected CBCT scans (approximately 14% of the total sample) were re-measured using the same segmentation parameters and measurement protocol applied in the primary analysis. Airway segmentation and measurement procedures are illustrated in Figure 1. The anatomical landmarks used for airway length and airway index measurements are illustrated in Figure 2.

Statistical analysis

Statistical analysis was performed using SPSS Statistics version 22 (IBM Corp., Armonk, NY, USA), a commercially licensed software accessed under the institution’s subscription. Descriptive statistics were calculated for all airway parameters and are presented as mean ± SD. Data normality for each airway variable was assessed using the Shapiro-Wilk test prior to inferential analysis. Based on the distribution of the data, comparisons across the 11 age groups were conducted using one-way analysis of variance (ANOVA) for normally distributed variables and the Kruskal-Wallis test for non-parametric variables, in accordance with established statistical approaches used in airway assessment studies [6,17]. A p-value < 0.05 was considered statistically significant.

Inter- and intra-observer reliability were evaluated to assess measurement precision and reproducibility using the Intraclass Correlation Coefficient (ICC) and the Dahlberg error statistic [22]. The ICC values were calculated using a two-way random-effects model with absolute agreement, while Dahlberg’s formula was applied to quantify method error. Mean reliability estimates with corresponding 95% confidence intervals were calculated for each airway measurement.

Reliability was interpreted according to established criteria, whereby ICC values ≥ 0.75 indicate good reliability and values ≥ 0.90 indicate excellent reliability. In CBCT-based airway morphometric studies, inter-observer ICC values ≥ 0.80 and intra-observer ICC values ≥ 0.90 are generally considered indicative of acceptable to excellent reproducibility. Method error was evaluated using Dahlberg’s formula, with lower values representing greater measurement precision; errors constituting a small proportion of the mean measurement are considered acceptable for both volumetric and linear airway assessments. Based on these established benchmarks, the reliability analysis confirmed that the airway measurements obtained in this study were reproducible and methodologically robust. Statistical analyses were limited to age-based comparisons and associations. Sex, body mass index, and craniofacial skeletal classification were not included as independent variables or covariates in this pilot study.

Correlation analysis between age and airway measurements

Correlation analysis was performed to evaluate the association between age and airway parameters, including airway volume, airway length, smallest cross-sectional area, and airway index. Depending on data normality, either Pearson’s or Spearman’s correlation coefficients were used to determine the strength and direction of these associations. This analysis enabled assessment of continuous age-related variations in airway morphology beyond simple intergroup comparisons. The correlations reflect associations within the study sample and do not imply causal or developmental effects.

Regression analysis

Multivariable regression analyses adjusting for sex, body mass index, or craniofacial skeletal classification were not performed, as these variables were not systematically recorded or analyzed in this pilot study. Accordingly, statistical evaluation was restricted to age-based intergroup comparisons and correlation analyses.

Ethical approval

This research was approved by the Institutional Ethics Committee of Karpaga Vinayaga Institute of Dental Sciences in Padalam, Tamil Nadu, India (approval no. KIDS/IEC/2024/II/003). All procedures were conducted in accordance with the principles of the Declaration of Helsinki on biomedical research involving human participants. Informed written consent was obtained from all adult participants. For minors under 18 years of age, consent was obtained from parents or legal guardians via a Google Form (Google LLC, Mountain View, CA, USA; see Appendix A for sample consent form). Participant confidentiality was maintained throughout the study. No additional radiation exposure was incurred for research purposes, as all CBCT scans were obtained for clinical indications.

## Results

Descriptive analysis of airway parameters in different age groups

The airway parameters were analyzed descriptively, and there was significant age variation. Table 1 indicates a continuous increase in airway volume, minimum airway area, and airway index between childhood and young adulthood. The group with the lowest values was the six- to 10-year-old age group, and a steep jump was observed between the 16- to 35-year-old group with the highest values. There was a small yet steady increase in airway length across all groups, but no significant difference. The decline in airway volume, minimal region, and index at the age of 36 years demonstrated the indication of major developmental alterations with age, and then a reduction in airway sizes with age.

**Table 1:** Descriptive statistics (mean, median, and SD) of airway parameters across age groups Values are presented as mean ± SD and median. Airway parameters were calculated using validated CBCT-based airway measurement protocols [1,7,15]. CBCT: Cone-beam computed tomography

Age group (in years)	Mean ± SD	Median (Q1-Q3)	Shapiro-Wilk p
Six to 10	15,108.7 ± 1,815.6	14,872.5 (14,519.5–15,724.0)	0.91
11 to 15	17,334.8 ± 959.1	17,297.0 (16,502.0–18,127.5)	0.46
16 to 20	18,995.8 ± 1,016.8	19,103.5 (18,617.3–19,705.0)	0.13
21 to 25	21,570.2 ± 1,361.6	21,282.5 (20,620.8–22,669.0)	0.91
26 to 30	24,192.5 ± 1,354.6	24,107.0 (23,265.0–25,180.5)	0.46
31 to 35	26,488.8 ± 1,544.4	26,205.0 (25,511.0–27,612.0)	0.05
36 to 40	29,734.3 ± 1,498.3	29,734.3 (28,920.0–30,480.5)	0.91
41 to 45	32,617.8 ± 1,437.2	32,617.8 (31,650.0–33,690.0)	0.42
46 to 50	33,518.0 ± 1,621.9	33,518.0 (32,410.5–34,690.0)	0.59
51 to 55	36,886.0 ± 1,449.1	36,886.0 (35,830.0–37,905.0)	0.60
56 to 60	39,812.5 ± 1,324.4	39,812.5 (38,920.0–40,760.0)	0.59

**Figure 3:**
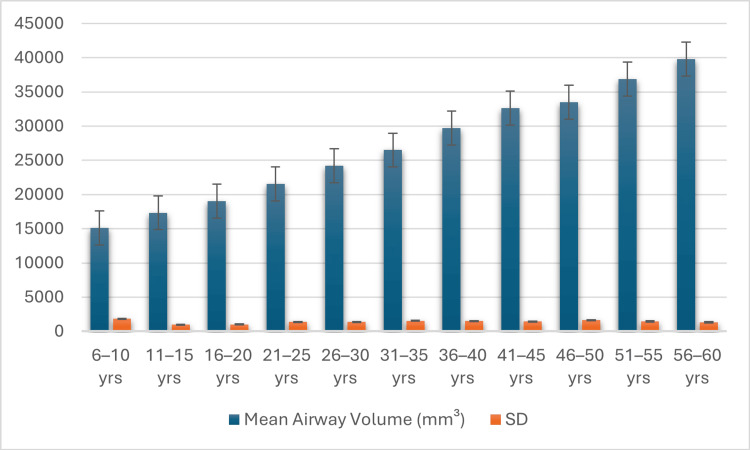
Mean airway volume across age groups Airway measurements were calculated using validated CBCT-based airway morphometric methods [1,7,15]. Graph created by authors. CBCT: Cone-beam computed tomography

The airway volume continued to increase with age in childhood and adulthood. The airway volume was lower in the younger age groups (six to 15 years), and there was a sharp rise in the age group of 16 to 35 years of age, as revealed in Figure 3.

The maximum mean values were observed in the 31 to 35 years group, which represents the maximum developmental stage of life. A constant decrease in the airway volume was then observed after 36 years, and the decrease increased after 40 years. The findings were that there was a significant effect of age, which demonstrated that the morphology of the airways changed considerably during the growth phases and declined slowly as age changed.

ANOVA across age groups

Both parametric and non-parametric statistical tests were applied to evaluate differences in upper airway measurements across the 11 independent age groups. Given that each age group contained a small sample size (n = 10), normality could not be assumed a priori. Therefore, distributional assumptions were evaluated individually for each airway variable using the Shapiro-Wilk test [23]. One-way ANOVA was used only for variables that demonstrated approximate normal distribution [6], followed by Tukey’s honestly significant difference (HSD) post hoc test to identify inter-group differences. For variables that did not meet normality assumptions, the Kruskal-Wallis test was applied [17], with post hoc comparisons performed using Dunn’s test.

No analysis of covariance (ANCOVA) was performed, as no covariates were included in the statistical models. This analytical approach ensured appropriate group-wise comparisons based on data distribution characteristics while maintaining consistency with the study methodology.

Comparison of the analysis of airway parameters by ANOVA

The one-way ANOVA comparison of airway parameters between the groups showed that there were significant differences between the age groups. The airway index, minimum airway area, and airway volume exhibited a strongly significant difference (p < 0.001), ideally portraying that there is a significant difference in these variables across various levels of development, as shown in Table 2. 

**Table 2:** Intergroup comparison of airway parameters using ANOVA Intergroup comparisons were performed using one-way ANOVA. A p-value < 0.05 was considered statistically significant. Airway volume, smallest airway area, and airway index demonstrated significant variations across age groups, whereas airway length remained relatively stable. ANOVA: Analysis of variance

Parameter	F-value	p-value	Significance
Airway volume	15.24	<0.001	Significant
Airway length	3.32	0.058	Not significant
Smallest area	14.11	<0.001	Significant
Airway index	16.35	<0.001	Significant

**Figure 4:**
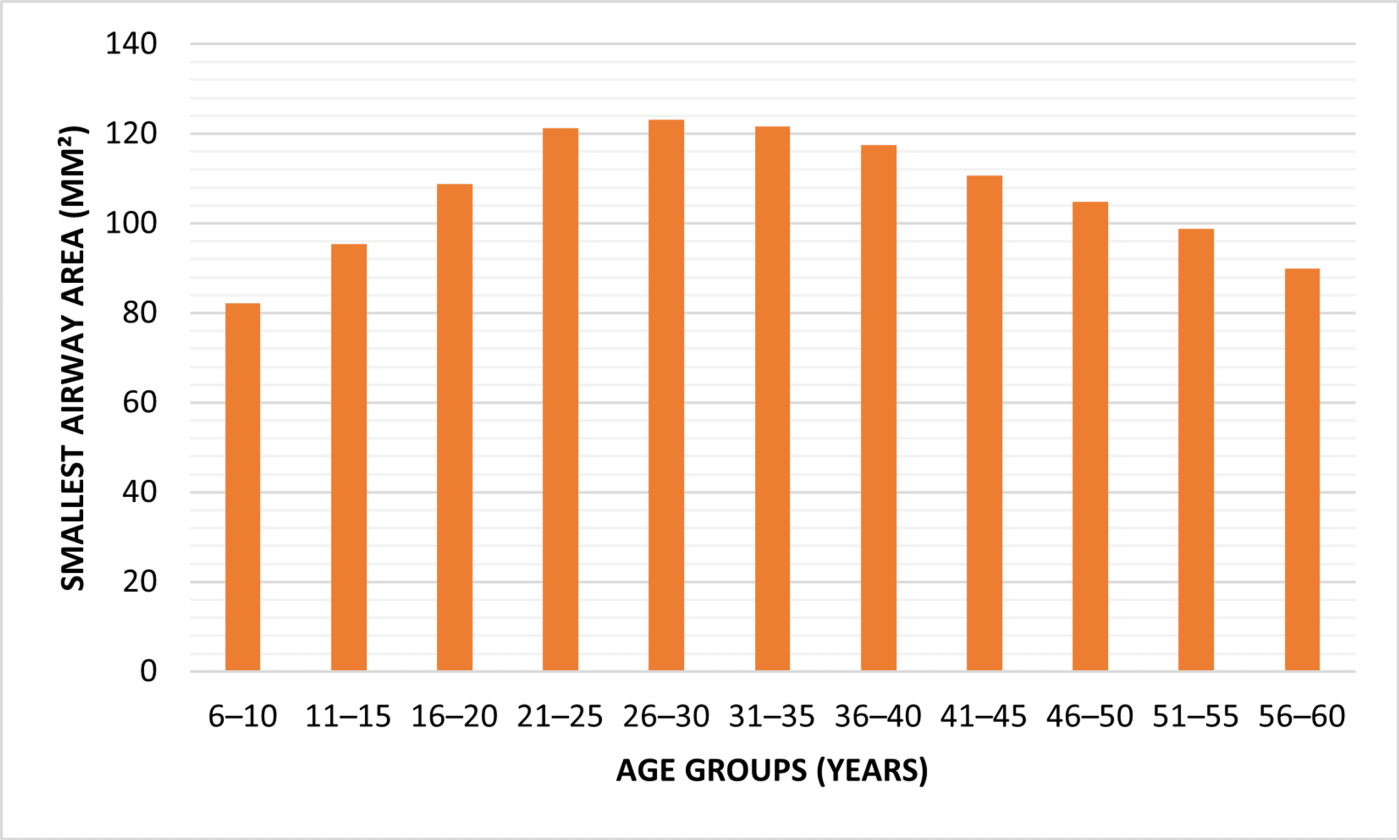
Smallest airway area across age groups Airway measurements were calculated using validated CBCT-based airway morphometric methods [1,7,15]. Graph created by authors. CBCT: Cone-beam computed tomography

The highest values were observed among the age group of 21 to 35 years, and the older groups showed a progressive decrease. Nevertheless, there was no statistically significant difference in airway length (p = 0.058), which can be interpreted as some relative stability across age groups. These observations emphasized that structural airway parameters, excluding airway length, were significantly driven by age-related growth trends and followed reductions identified with increasing age. The smallest cross-sectional airway area showed a consistent increase during the initial stages of growth. Children between the ages of six and 10 years showed the least mean area values, while there was a steady increase up to the 26- to 30-year-old age group (Figure 4).

Maximal values were found in the range of 26 to 35 years, showing the stage of maximum airway expansion. A gradual diminution in the smallest airway area after the age of 36 was found, showing age-related changes in airway morphology. These findings validated that growth-related structural changes happened predominantly during young adulthood and adolescence, followed by progressive reduction in the airway dimensions throughout middle and older ages.

Correlation analysis between airway parameters and age

Simple linear regression analysis was performed to evaluate the association between age (independent variable) and each airway measurement (dependent variable), to provide a more specific and comparable assessment than correlation analysis. The regression analysis demonstrated significant positive associations between age and airway volume (standardized β = +0.74, p < 0.001), smallest airway cross-sectional area (standardized β = +0.69, p < 0.001), and airway index (standardized β = +0.76, p < 0.001), indicating progressive increases in these airway dimensions up to early adulthood.

Airway length showed a weak and non-significant association with age and was therefore not a significant predictor in the regression model. Standardized regression coefficients were reported to allow direct comparison of effect magnitude across airway variables. Importantly, these regression coefficients reflect cross-sectional associations observed within this study sample and should not be interpreted as evidence of causal, developmental, or longitudinal effects of age on airway morphology. In addition, the magnitude of the observed effect sizes should be interpreted cautiously, given the limited number of participants within each age group in this pilot study. The results of the regression analysis are presented in Table 3.

**Table 3:** Bivariate linear regression analysis of airway parameters with age Pearson’s correlation test was used. Airway volume, smallest airway area, and airway index showed a strong positive correlation with age up to 35 years, followed by a gradual decline thereafter. Airway length showed a weak, non-significant correlation.

Parameter	Standardized regression coefficient (β)	p-value	Interpretation
Airway volume	+0.74	<0.001	Significant positive association with age
Airway length	+0.28	0.065	No statistically significant association
Smallest airway area	+0.69	<0.001	Significant positive association with age
Airway index	+0.76	<0.001	Significant positive association with age

**Figure 5:**
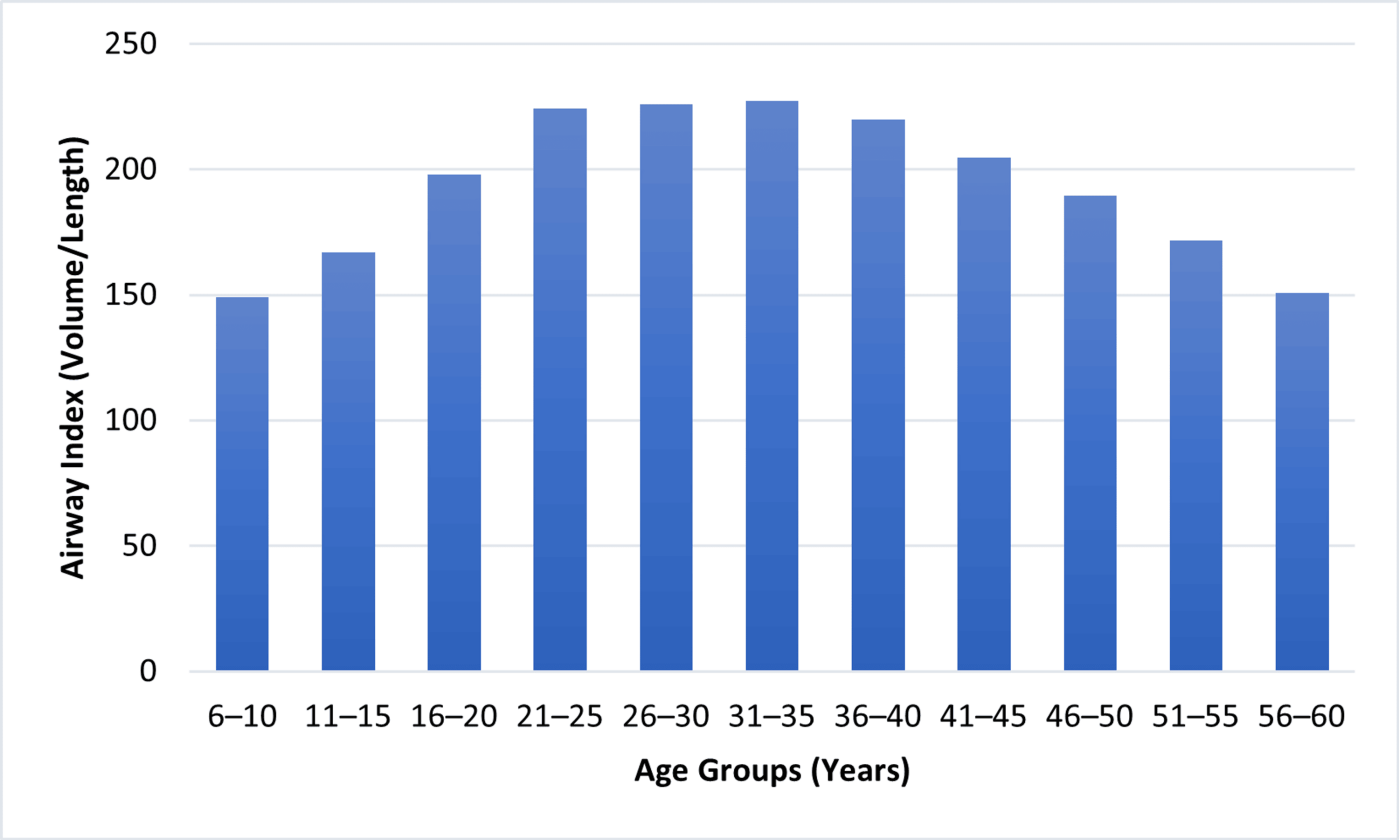
Airway index across age groups Airway measurements were calculated using validated CBCT-based airway morphometric methods [1,7,15]. Graph created by authors. CBCT: Cone-beam computed tomography

There was a weak (statistically non-significant) relationship between the airway’s length (r = +0.28, p = 0.065) and relative stability across the age groups. These findings indicate that, based on bivariate linear regression analysis, age showed a statistically significant association with airway volume, smallest airway cross-sectional area, and airway index, whereas airway length did not demonstrate a significant association with age. Airway index in relation to airway volume over airway length expressed progressive tendencies across study groups. The measurements at an early age (six to 15 years) had a relatively lower value, which increased abruptly in adolescence and early adulthood (Figure 5).

The highest airway index values were observed in the 26- to 35-year age group. Comparisons across independent age groups showed lower mean values in both younger and older age categories. However, these differences represent cross-sectional associations between age groups rather than individual developmental change.

## Discussion

This study employed one-way ANOVA to compare airway parameters across independent age groups and bivariate linear regression analysis to evaluate the association between age as a continuous variable and airway dimensions. The data analysis indicated that there were age differences in the air and structural differences in the airways. Airway volume, minimum airway area, and airway index all increased with age, with a maximum at age 21 to 35 years, and then fell steadily over the next decades (Table 1, Figures 3-5). Airway length, by contrast, remained fairly constant between groups. Intergroup ANOVA identified statistically significant differences among volume, smallest area, and index (all p < 0.001), but not for length (Table 2). Bivariate linear regression analysis showed that age was significantly associated with airway volume, smallest airway cross-sectional area, and airway index, whereas airway length did not demonstrate a statistically significant association with age (Table 3). These findings represent cross-sectional associations observed between independent age groups and should not be interpreted as evidence of individual-level growth, decline, or biological aging effects over time.

The present findings are consistent with previous cross-sectional CBCT studies that have reported variations in airway length and size across different age groups in cohorts spanning from childhood to older adulthood. These observations reflect between-group differences rather than longitudinal increases, plateaus, or decreases within the same individuals [21]. Parallel findings have been reported in recent cross-sectional CBCT studies, which described differences in oropharyngeal airway volume across age groups, reflecting between-group variation rather than individual-level loss or age-related decline over time [24]. Previous studies have reported associations between age and airway size in specific populations, such as children and adolescents with defined skeletal classifications; however, the present study did not evaluate skeletal maturation or skeletal class, and its findings are limited to age-based associations within the study sample [25]. Previous interventional and longitudinal studies have reported that maxillary expansion is associated with changes in airway volume in selected patient groups; however, the present cross-sectional study did not evaluate treatment effects or within-individual change, and the observed differences reflect comparisons between independent age groups rather than expansion-related increases [6,26]. Finally, a normative CBCT dataset study established that both gender and age were important in determining airway morphology and the pattern of volume changes with age [27]. While the literature suggests patterns of airway growth and reduction across age groups, such findings consistently represent population-level or cross-sectional associations rather than causal or developmental trajectories [28]. The inclusion of median values provided complementary descriptive information to the mean ± SD, allowing a more balanced summary of the distribution of airway measurements within the study sample, rather than validating the estimates as population-level values. The trends of both measurements were similar, and significant changes in airway volume, area, and index increased until early adulthood (21 to 35 years) and then declined subsequently with age. These descriptive trends should not be interpreted as longitudinal changes or normative developmental patterns.

The clinical relevance of the present findings lies in their ability to describe cross-sectional differences in airway measurements across independent age groups within the study sample. Rather than establishing normative reference standards or diagnostic thresholds, the results provide descriptive information that may assist clinicians in contextualizing CBCT-derived airway measurements when evaluating patients of different ages. The observed variation in airway volume, smallest airway area, and airway index across age groups highlights that airway dimensions differ between younger, middle-aged, and older individuals; however, these differences may be influenced by unmeasured confounding factors and should not be interpreted as evidence of individual growth, decline, or age-related risk.

Accordingly, the findings may support hypothesis generation and encourage cautious consideration of age-related variability during orthodontic assessment and treatment planning, without implying prediction, screening, or diagnosis of airway-related conditions such as obstructive sleep apnea. Future longitudinal and population-based studies incorporating clinical outcomes, skeletal classification, BMI, and functional respiratory assessments are required before age-specific airway measurements can be translated into diagnostic, prognostic, or treatment-guiding applications.

This study should be interpreted primarily within the context of its methodological limitations. As a cross-sectional pilot investigation conducted at a single academic center with a limited sample size (n = 110), the findings are descriptive and exploratory in nature and cannot be generalized to the broader population. Although equal age stratification allowed structured comparisons between independent age groups, the design does not permit assessment of individual-level change, developmental trajectories, or causal relationships. The CBCT-based 3D airway measurements were performed using standardized software and protocols, and intra- and inter-observer reliability assessment demonstrated acceptable measurement reproducibility; however, these reliability measures reflect consistency of measurement rather than biological validity or population representativeness.

Several variables known to influence airway dimensions, including sex distribution, BMI, skeletal maturity, and craniofacial classification, were neither quantitatively defined nor incorporated into the analytical models. Although such factors were considered during participant selection to exclude overt pathology, their effects could not be measured, adjusted for, or estimated in the statistical analysis. As a result, the observed differences in airway dimensions across age groups may be partially or substantially influenced by these unmeasured confounders rather than age alone. The inability to control for these factors limits the interpretation of the magnitude, direction, and specificity of age-related associations and precludes the attribution of observed airway differences to developmental or biological effects of aging. Given these limitations, the present findings should be viewed as hypothesis-generating rather than confirmatory. Future studies employing population-based sampling, clearly defined confounder stratification, and longitudinal follow-up are required before age-related airway differences can be translated into normative references or clinically actionable interpretations.

To extend these pilot results, future studies should include larger, multi-center cohorts to maximize representativeness and statistical power. Longitudinal follow-up would define individual growth trajectories of airway dimensions. Incorporation of artificial intelligence-assisted segmentation and volumetric analysis could simplify measurements and facilitate automated normative mapping. Sex stratification, BMI groups, and craniofacial phenotypes such as malocclusion classes could reveal different developmental patterns. Moreover, investigation of functional associations, such as sleep-disordered breathing risk in association with airway volume reduction, could improve clinical criteria. Generally, larger datasets and state-of-the-art imaging analytic technology will improve knowledge of airway development and age-related change for both research and clinical applications.

## Conclusions

This cross-sectional pilot study used CBCT-based computerized volumetric analysis to describe 3D airway measurements across independent age groups in a sample of participants from Tamil Nadu, India. Differences in airway volume, smallest airway cross-sectional area, and airway index were observed between age groups, while airway length showed relatively limited variation. These findings, however, represent cross-sectional associations rather than individual-level change or developmental progression. Given the study design, convenience sampling, single-center setting, and limited sample size, the results should be interpreted as descriptive and exploratory and do not support the establishment of normative reference values, causal inferences, or prediction of clinical outcomes. In addition, several variables known to influence airway dimensions, including sex distribution, BMI, skeletal maturity, and craniofacial classification, were not quantitatively defined or incorporated into the statistical analyses, further limiting generalizability and clinical applicability. Accordingly, the findings should not be used to guide diagnosis, treatment planning, risk assessment, or screening for airway-related conditions. Instead, the present results provide preliminary information on age-group variability in CBCT-derived airway measurements and may serve as a foundation for hypothesis generation. Future population-based, multicentric, and longitudinal studies with clearly defined covariates, repeated measurements, and functional respiratory outcomes are required before age-related airway differences can be translated into normative frameworks or clinically actionable interpretations.

## Appendices

Appendix A

Informed Consent for CBCT Scan and Use in Retrospective Study

This form records informed consent for a diagnostic CBCT scan performed for clinical purposes. You are also consenting to allow anonymized scan data to be used in a retrospective study approved by the Institutional Ethics Committee at Karpaga Vinayaga Institute of Dental Sciences.
 

Full Name of the Child / Minor Participant: _______________________________
 

Age of the Child at the Time of Scan: _______________________________
 

Full Name of Parent / Legal Guardian: _______________________________
 

Relationship to the Child: _______________________________
 

Patient ID: _______________________________
 

1. I understand that the CBCT scan is being conducted for diagnostic or treatment purposes.  ☐ Yes ☐ No ☐ Maybe

2. I voluntarily give my consent for the CBCT scan to be performed on my child.  ☐ Yes ☐ No

3. I agree to allow the anonymized CBCT scan data to be used in this ethics‑approved research study.  ☐ Yes ☐ No ☐ Only if identity is protected

4. I understand that no additional procedures or radiation exposure will be performed for research purposes.  ☐ Yes ☐ No

5. I understand that participation in this study is voluntary and refusal will not affect clinical care.  ☐ Yes ☐ No

6. I understand that I can withdraw consent at any time before data usage.  ☐ Yes ☐ No

7. I confirm the CBCT scan is performed in a registered dental hospital or teaching institute.  ☐ Yes ☐ No

Signature of Parent / Guardian: _________________________

Name: _________________________

Date: _________________________
